# Vesicular Stomatitis Virus: Insights into Pathogenesis, Immune Evasion, and Technological Innovations in Oncolytic and Vaccine Development

**DOI:** 10.3390/v16121933

**Published:** 2024-12-18

**Authors:** Mohamed Mustaf Ahmed, Olalekan John Okesanya, Bonaventure Michael Ukoaka, Adamu Muhammad Ibrahim, Don Eliseo Lucero-Prisno

**Affiliations:** 1 Faculty of Medicine and Health Sciences, SIMAD University, Mogadishu 252, Somalia; 2 Department of Medical Laboratory Science, Neuropsychiatric Hospital, Aro, Abeokuta 110101, Nigeria; okesanyaolalekanjohn@gmail.com; 3 Community and Clinical Research Division, First On-Call Initiative, Port Harcourt 500001, Nigeria; bonaventureukoaka@gmail.com; 4 Department of Immunology, School of Medical Laboratory Science, Usmanu Danfodiyo University, Sokoto 840001, Nigeria; amuhammadibrahim37@gmail.com; 5 Department of Global Health and Development, London School of Hygiene and Tropical Medicine, London WC1E 7HT, UK; luceroprisno@gmail.com; 6 Research and Innovation Office, Southern Leyte State University, Leyte 6500, Philippines; 7 Research and Development Office, Biliran Province State University, Biliran 6549, Philippines

**Keywords:** vesicular stomatitis virus, oncolytic virotherapy, vaccine vector, pathogenesis, immune evasion

## Abstract

Vesicular stomatitis virus (VSV) represents a significant advancement in therapeutic medicine, offering unique molecular and cellular characteristics that make it exceptionally suitable for medical applications. The bullet-shaped morphology, RNA genome organization, and cytoplasmic replication strategy provide fundamental advantages for both vaccine development and oncolytic applications. VSV’s interaction with host cells through the low-density lipoprotein receptor (LDL-R) and its sophisticated transcriptional regulation mechanisms enables precise control over therapeutic applications. The virus demonstrates remarkable versatility through its rapid replication cycle, robust immune response induction, and natural neurotropism. Recent technological innovations in VSV engineering have led to enhanced safety protocols and improved therapeutic modifications, particularly in cancer treatment. Attenuation strategies have successfully addressed safety concerns while maintaining the therapeutic efficacy of the virus. The molecular and cellular interactions of VSV, particularly its immune modulation capabilities and tumor-selective properties, have proven valuable in the development of targeted therapeutic strategies. This review explores these aspects, while highlighting the continuing evolution of VSV-based therapeutic approaches in precision medicine.

## 1. Introduction

The application of viruses in precision and therapeutic medicine has advanced significantly in the recent decades. From its use in vaccine development to oncolytic virotherapy and genetic therapeutics, this development has emerged as a vital tool for medical evolution [1,2]. Vesicular stomatitis virus (VSV) is widely known as the implicated microbe for vesicular stomatitis (VS), which has historically been recognized as a significant veterinary pathogen that causes vesicular stomatitis, an acute viral disease that primarily affects cattle, horses, and swine [3]. The virus’s simple molecular structure, rapid replication cycle, and well-characterized biology have made it an invaluable model system for fundamental studies in molecular virology and cell biology [4]. In recent decades, VSV has emerged as a promising platform for vaccine development and therapeutic applications owing to its ability to trigger robust immune responses and its adaptability as a vector system [4].

VSV belongs to the Rhabdoviridae family, which includes the genera Vesiculovirus, Lyssavirus, and Ephemerovirus, which primarily infect animals, and Cytorhabdovirus and Nucleorhabdovirus, which primarily target plants. Lyssavirus is best known for containing the rabies virus, whereas VSV is the most common representative of the Vesiculovirus genus [5]. There are two main serotypes of VSV, VSV-New Jersey (VSV-NJ) and VSV-Indiana (VSV-I), which differ in geographic distribution and virulence, with VSV-I further classified into serological subclasses [6]. Structurally, VSV has a bullet-shaped morphology and an RNA genome encoding five major proteins: nucleoprotein (N), phosphoprotein (P), matrix protein (M), glycoprotein (G), and viral polymerase (L) [6]. Viral entry and exit, as well as cellular recognition and fusion, are facilitated by the G protein, whereas the M protein is essential for viral assembly and inhibits host mRNA export [7]. Cell transcription is performed by RNA-dependent RNA polymerase (RdRp), which is induced by P and L proteins [8].

Since the late 20th century, VSV has been a major molecular virological microbe of research interest. Unlike DNA viruses, which are historically used as vaccine vectors and oncolytic agents, RNA viruses, such as VSV, are now being explored because of their potential advantages over DNA viruses in overcoming limitations related to genome integration risks [2]. With its RNA genome, VSV can undergo genetic replication within the cytoplasm. VSV produces five distinct subgenomic messenger RNAs, each of which encodes one of the five unique viral proteins [2]. The 11-kilobase (kb) genome, a single RNA strand of negative polarity, is entirely coated with the viral nucleoprotein [2].

Economically, while VSV infections rarely result in high mortality, they lead to production losses in livestock owing to movement restrictions and quarantines aimed at preventing the spread of the disease [6]. With advances in recombinant technology, modified VSV has been explored in vaccine development, most notably in the Ervebo vaccine for Ebola, which uses a live attenuated VSV vector [9,10]. Additionally, VSV has shown promise in oncolytic virotherapy due to its ability to preferentially infect and kill cancer cells deficient in the type 1 interferon (IFN-1) pathway, sparing normal tissues [11,12]. The selective replication of the virus in malignant tissues has opened new avenues for cancer treatment, especially against cancers such as glioblastoma [12]. Some oncolytic VSV strains express immunostimulatory cytokines, such as VSV-IL-4, whereas others incorporate suicide genes [13,14]. Similarly, VSV-CD/UPRT expresses cytosine deaminase and uracil phosphoribosyltransferase, leading to the destruction of infected bystander cells when treated with 5-fluorocytosine [14]. This highlights the importance of an age-long virus in modern medical technology. Despite this progress, knowledge gaps remain concerning the mechanisms of VSV pathogenesis, especially its immune evasion strategies and host defense interactions. As ongoing technological innovations emerge, these advances may further enhance VSV’s safety and efficacy in therapeutic applications. This study provides an in-depth review of current insights into VSV pathogenesis and immune evasion mechanisms. Additionally, this review discusses the latest developments in its applications in oncolytic virotherapy and vaccine design.

## 2. Materials and Methods

### 2.1. Search Strategy

The literature used in this review was obtained through an online search across multiple electronic databases: PubMed, Scopus, and Google Scholar. PubMed searches utilized Medical Subject Headings (MeSH) terms. Keywords such as “vesicular stomatitis”, “vesicular stomatitis virus”, “VSV”, “oncolytic viruses”, and “vesiculovirus” were combined in the search string. Boolean operators (AND/OR) were used to identify and streamline the vital phrases. An additional search of the reference lists of the included papers provided additional studies for review. The search was not restricted to any period in order to acquire a comprehensive review of the work performed in the field. A comprehensive literature search was conducted between 4 August 2024 and 10 September 2024.

### 2.2. Inclusion and Exclusion Criteria

Studies were considered for review if they satisfied the following criteria: published in full-text format in the English language, discussed the use of VSV for vaccine development or oncotherapy, published in a peer-reviewed journal, and used an experimental or quasi-experimental design along with reviews. Consequently, studies that were not within the scope outlined were excluded from the review.

### 2.3. Study Selection Process and Data Extraction Analysis

Two authors (MMA and OJO) independently evaluated the eligibility of selected studies using a rigorous screening process. Despite the limitation of having two reviewers, both authors dedicated substantial time and effort over seven weeks to thoroughly screen titles and abstracts, focusing specifically on content relevant to VSV applications in vaccine development and therapeutic approaches. Regular periodic consensus discussions were held between the authors to ensure consistency in the selection process and to resolve any discrepancies by consulting additional authors (AMI). Data were extracted from selected studies. The extracted information underwent content analysis and the identified themes were subsequently discussed. Trends were identified and a thorough evidence synthesis was performed to compile the review.

## 3. Vesicular Stomatitis Virus (VSV) Structure and Serotypes

**Figure 1:**
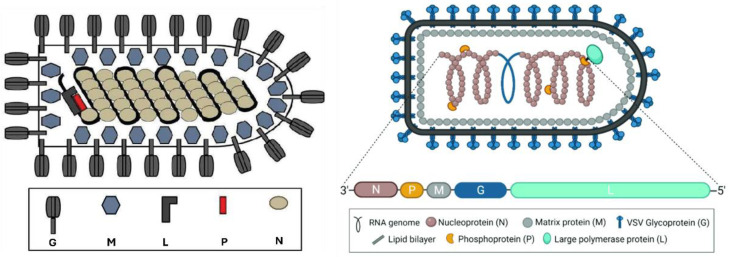
(**Left**) Schematic structure of VSV, consisting of a nucleocapsid core with RNA, N protein, L, and P protein complexes, and an envelope with G protein and M protein inner surfaces [32]. (**Right**) The VSV and its genomic structure show the characteristics of bullet-shaped, negative-sense, single-stranded RNA encapsulated by the nucleoprotein (N), phosphoprotein (P), and RNA-dependent RNA polymerase (L). The matrix protein (M) condenses the nucleocapsid, driving virion budding, whereas the glycoprotein (G) studs the surface of the virion and exists in trimeric complexes [4].

VSVs are enveloped, non-segmented, negative-stranded RNA arthropod-borne viruses, also known as arboviruses (Figure 1), which have a characteristic bullet-shaped virion structure and range in size from 65 to 185 nm [15]. VSV, the prototype virus of the Rhabdoviridae family, has been thoroughly investigated in vitro, mostly to investigate the mechanisms underlying interferon generation [15,16]. New Jersey virus (VSNJV) and Indiana virus (VSV), the latter of which is further subdivided into three serological groups, are the two main serotypes of VSV. The classical strain of Indiana is Indiana type 1, whereas the prototype viruses of Indiana subtypes 2 and 3 are Cocal virus (COCV) and Alagoas virus (VSAV), respectively [17,18]. While VSAV was first isolated from a mule in Brazil in 1964, COCV was first isolated in the early 1960s from mites collected from rice rats in Trinidad and Northern Brazil [19]. Other vesiculoviruses that can cause lesions in domestic animals during experimental inoculation include the Piry virus, Chandipura virus, and Isfahan virus [19,20].

Similar to all rhabdoviruses, the VSV virion comprises two main parts: an internal ribonucleoprotein core and an exterior envelope. Based on the membrane of the host cell, the envelope carries the viral glycoprotein, also known as the G protein, which is an essential transmembrane protein that generates approximately 400 trimeric spikes. Membrane-bound ribosomes manufacture the G protein, and calnexin and BiP act as chaperones to aid proper folding. The protein is glycosylated and acylated as it passes through the Golgi apparatus and travels to the cell membrane [21,22]. The G protein is necessary for cell fusion and recognition and is also a key factor in defining the specificity of neutralizing antibodies. Notably, pH-dependent infectivity mediated by the G protein is linked to the virulence of distinct serotypes, such as the greater virulence of New Jersey compared to Indiana [23,24]. The viral genome is contained within the N protein and comprises the internal nucleocapsid core. Along with viral RNA, these N proteins are organized into a “beads-on-a-string” structure to produce an RNase-resistant core [22]. The large (L) and phosphoprotein (P) proteins comprise the viral RNA-dependent RNA polymerase, which is necessary for the replication of VSV because it is a negative-stranded RNA virus [25,26]. The N-protein–RNA complex interacts with the P-L complex during viral transcription and replication. When the P protein forms trimers, it helps the L protein and the N-RNA complex bind together to generate the active transcriptase required for viral replication [27,28]. Another essential element is the matrix protein (M), which is located between the nucleocapsid core and the interior surface of the viral envelope. M protein is involved in several processes such as nucleocapsid condensation during viral assembly and viral particle budding [29,30]. The transmembrane G protein can vary significantly between and within distinct VSV serotypes, which is noteworthy because this variability in the G protein influences the ability of the virus to attach to host cells and evade immune responses, potentially affecting VSV’s infectivity and adaptability. In contrast, the viral N, L, P, and M proteins maintain a constant composition among viral particles, ensuring stability in the core structure and function of the virus [22,31].

## 4. VSV Entry, Fusion, and Replication Mechanisms

### 4.1. Entry and Receptor Recognition

VSV interacts with the cell surface and enters the endocytic pathway. The primary cellular receptor for VSV is the low-density lipoprotein receptor (LDLR), which serves as the key entry point for viral attachment and subsequent infection [33]. Owing to the ubiquitous expression of LDL-R across different cell types and species, VSV glycoprotein (VSV-G) demonstrates remarkable versatility in cellular entry [33]. This broad tropism has made VSV-G particularly valuable in biotechnological applications, where it is frequently used to pseudotype other viruses, particularly lentiviruses, to enhance their entry efficiency and broaden their host range.

### 4.2. Endocytosis and Membrane Fusion

VSV can initiate signaling pathways within cells, like other viruses, which helps it to be taken up by the host cell and used as an invasion vector (Table 1). The virus enters cells through a self-initiated endocytic process that requires actin polymerization [34,35]. This process can be inhibited by compounds such as cytochalasin D or latrunculin B, which prevent virus-containing pits from developing into vesicles [21,36]. Following endocytosis, the viral nucleocapsid is released into the cytosol within two minutes of fusion with the early endosomes [37]. The virus enters cells through a self-initiated endocytic process that requires actin polymerization. This process can be inhibited by compounds such as cytochalasin D or latrunculin B, which prevent virus-containing pits from developing into complete vesicles [21,36]. Following endocytosis, the viral nucleocapsid is released into the cytosol within two minutes of fusion with the early endosomes [37]. A drop in pH (below 6.5) triggers a conformational change in the G protein, enabling fusion between the viral envelope and the endosome membrane. This fusion event releases the nucleocapsid into the cytoplasm [38]. Research using RAB GTPases has shown that early endosomes are the primary sites of VSV fusion, as demonstrated by the inhibitory effect of RAB 5 mutants on infection [39].

### 4.3. Transcription and Replication

Following successful entry and uncoating, VSV begins its replication cycle, which is typical for negative-stranded RNA viruses. The viral genome, encoded by nucleoprotein (N), serves as a template for initial transcription. The RNA-dependent RNA polymerase (L protein) of the virion catalyzes this process by synthesizing leader RNA and mRNAs for the five viral proteins: N, P, M, G, and L [40,41]. Viral RNA polymerase, working with P protein trimers that bind to the L protein, carries out genome transcription [39,42]. This process begins at the 3′ end of the genome with the synthesis of a 47-nucleotide leader RNA, followed by transcription of viral mRNAs [38]. The early steps of the replication cycle occur within the first few hours of infection, while the later stages take 12–18 h to complete.

Research using RAB GTPases has demonstrated that while mutants impacting traffic to late endosomes or recycling endosomes have no such effect, an RAB 5 mutant, which slows endocytic vesicle maturation, can impede VSV infection. This implies that early endosomes are the primary sites of VSV fusion [39]. Another hypothesis with less evidence supports the idea that VSV releases its nucleocapsid in two stages. Within multivesicular bodies (MVBs), the viral envelope fuses with an internal vesicle. This is followed by internal vesicle back-fusion with the MVB-limiting membrane [31]. The finding that VSV fusion is favored by bis (monoacylglycerol)phosphate (BMP), a lipid unique to interior MVB vesicles, lends credence to this theory. Although it is still feasible that VSV may use various entrance methods depending on the physiological state of the host cell, the majority of available data support the classical notion of fusion occurring in early endosomes [31,43].

VSV has a replication cycle typical of negative-stranded RNA viruses. After the virus attaches to the host cell, it penetrates the membrane, uncoats it, and releases its nucleocapsid into the cytoplasm. The viral genome, encoded by nucleoprotein (N), serves as a template for initial transcription. The virion’s RNA-dependent RNA polymerase (L protein) catalyzes this process by synthesizing leader RNA and mRNAs for five viral proteins: N, phosphoprotein (P), matrix protein (M), glycoprotein (G), and large polymerase protein (L) [40,41]. These primary transcripts are essential for producing viral proteins for genome replication and assembly. Viral RNA polymerase synthesizes full-length positive-strand RNA (antigenome), which is used as a template for the viral genome. The early steps of the replication cycle occur within the first few hours of infection, while the later stages take 12–18 h to complete. Phosphatidylserine, a potential receptor for VSV, is unlikely to localize to the plasma membrane, making it inaccessible for entry. Instead, VSV may attach to host cells through nonspecific electrostatic and hydrophobic interactions facilitated by a lower pH, which is crucial for viral entry and membrane fusion [38]. A drop in pH triggers a conformational change in the G protein, allowing fusion between the viral envelope and the endosome membrane. The endosome, formed when the pH drops below 6.5, allows the G protein to mediate fusion between the viral envelope and the membrane, releasing the nucleocapsid into the cytoplasm. M protein dissociates from the nucleocapsid, allowing viral RNA synthesis. The viral genome is transcriptionally carried out by viral RNA-dependent RNA polymerase, with the P protein forming trimers that bind to the L protein [42,44]. Transcription starts at the 3′ end of the genome, followed by synthesis of a 47-nucleotide leader RNA and transcription of viral mRNAs for protein production [38].

## 5. VSV Host Cell Manipulation, Assembly, and Budding

VSV utilizes endocytic and secretory routes for its entry and replication cycles. Factors involved in clathrin-mediated endocytosis (CME) facilitate VSV entry into host cells. Once inside the cell, viral replication depends on the G protein being transported via the secretory route [45]. The vesicular transport of proteins from the ER to the Golgi apparatus, and ultimately to the plasma membrane, is mediated by coat protein complexes (COPI and COPII). Newly generated G proteins are initially transported by COPII from the ER to the COPII-coated vesicles [46,47]. After these vesicles combine to form larger complexes, COPI controls and guides the vesicles to the Golgi apparatus. Furthermore, COPI’s participation in viral gene expression is linked to VSV RNA production [48].

VSV uses complex mechanisms in the later phases of its replication cycle to control the host cell machinery. An essential aspect of this process is the capacity of the virus to subvert the host’s translation machinery and ensure preferential synthesis of its mRNAs [49]. Through a cap-dependent translation process, VSV uses the host machinery but strategically interferes with prioritizing its mRNA translation [50]. VSV specifically alters the eIF4F complex, a crucial host regulator of cap-dependent translation, by dephosphorylating the translation inhibitor 4E-BP1 and cap-binding protein eIF4E. This reduces the availability of active eIF4F complexes, enabling preferential translation of viral mRNAs to host mRNAs [38,51]. The virus can manipulate the cellular translation machinery to ensure that its mRNAs are preferentially translated while host mRNA translation is repressed, even if the precise processes underlying these alterations remain unclear (Table 2) [52]. The structural components of VSV mRNAs, which improve their translation efficiency, also help with this. These components could interact with ribosomal proteins, such as rpL40, to help construct the 80S ribosome, which is required for the translation of viral mRNA. It is noteworthy that rpL40 does not appear to be necessary for cap-dependent or internal ribosome entry site-driven mRNA translation in the host, but it appears to be necessary for the translation of mRNAs from different mononegavirales, such as measles, rabies, and VSV viruses [53,54]. To further control host cell activities, the VSV M protein inhibits host transcription and translation by interacting with nuclear export proteins, specifically Rae1 and Nup98, thereby preventing the transport of host transcripts from the nucleus to the cytoplasm [55]. Additionally, the M protein disrupts cellular transcription by deactivating the basal transcription initiation factor TFIID and downregulating RNA polymerase II [56]. Evidence suggests that the M protein may target the TATA-binding protein in TFIID, although the exact mechanism requires further investigation [57]. A viral replicase complex composed of P and L proteins is responsible for mediating replication of the VSV genome. In this process, the host variables also play a role. For example, the cytoskeleton protein tubulin may aid in viral genome replication by interacting with the L protein, and the chaperone protein HSP90AB1 improves the stability of VSV RNA-dependent RNA polymerase (RdRp) [55]. As it connects with the plus-strand and some minus-strand leader RNAs during infection, the La protein, which is typically linked to pol III transcripts, has also been linked to VSV RNA replication. Although further research is needed to determine the precise function of the La protein in genome replication, its involvement is correlated with increased replication efficiency [25,58].

**Figure 2:**
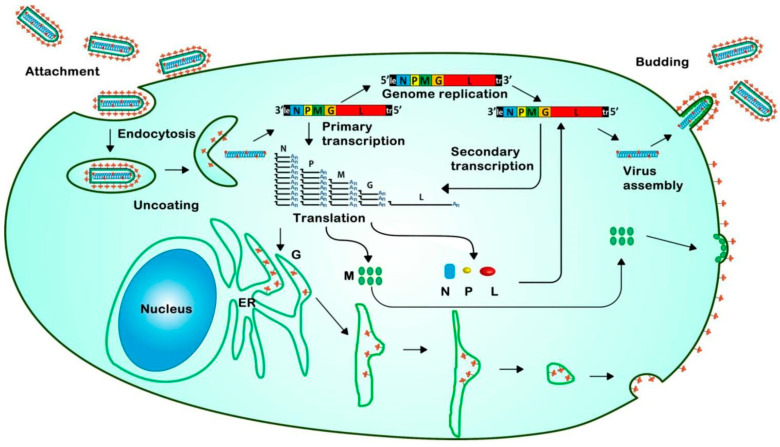
Schematic representation of the VSV life cycle showing key stages: attachment, endocytosis, uncoating, primary transcription, translation of viral proteins, genome replication through secondary transcription, virus assembly, and budding [69].

Viral M and G proteins mediate VSV assembly and budding at the plasma membrane, which follows genome replication. The host secretory pathway is used to produce G protein in conjunction with the endoplasmic reticulum (ER), where it undergoes glycosylation before being delivered to the plasma membrane through the Golgi apparatus [59]. This transport is facilitated by several cellular components, such as trafficking protein particle complex subunit 3 (Bet3), COPI, and COPII. It also requires the vesicle-trafficking protein SEC22b, which functions in the ER-Golgi transport intermediate compartment [60,61]. Meanwhile, the soluble M protein is brought to the plasma membrane by an unknown mechanism. Nonetheless, microdomains are formed as a result of their interaction with the plasma membrane, without the need for the G protein. These microdomains aid in the construction of the viral nucleocapsid, which is subsequently carried to the plasma membrane by a microtubule-dependent process and is associated with the M protein [55]. Interactions between the M protein and several cellular proteins are responsible for the last phases of VSV assembly, budding, and release. In its N-terminal region, the M protein has a PPxY motif that interacts with proteins with WW domains, such as Yes-kinase-associated protein (YAP), helping to draw these proteins to the budding site [62,63]. Furthermore, M protein-dynamin protein (dynamin 1 and 2) interactions facilitate viral budding and assembly (Figure 2). The machinery of cellular endosomal sorting complexes required for transport (ESCRT) is essential for the scission of the viral membrane from the host plasma membrane. ESCRT is involved in the synthesis of multivesicular bodies (MVBs) [55,64]. While VSV budding seems to require the ESCRT-I component TSG101, other components, such as VPS4A and 4 B, are required for this process [65]. Virus budding is usually tightly coupled to virion assembly, and most viruses use their structural proteins to recruit the ESCRT pathway [66]. Furthermore, as triacylglycerol production has been demonstrated to obstruct virus maturation, the lipid composition of the host cell membrane may affect M protein binding during this stage of viral development [55]. Notably, other host proteins, including integrin β1, HSP90, annexin 2, and EEF1A1, bundled within the virions were identified using mass spectrometry analysis of VSV virions. These proteins may be involved in virus assembly, budding, or other phases of the infection process; however, more research is needed to determine the precise roles of these proteins in the VSV life cycle [67]. Ultimately, VSV interacts with host cellular machinery in a very complex manner, using a variety of host pathways and proteins to guarantee proper assembly, release, and replication while disabling host defenses to promote viral spread [55,68].

## 6. Host Pathways Involved in VSV Replication and Immune Evasion

Host cells employ multiple defensse mechanisms against VSV infection, primarily through innate immune responses and cellular survival pathways. Understanding these host–pathogen interactions is crucial for the development of therapeutic applications. Innate immunological signaling pathways serve as primary defenses against VSV infection. Through pattern recognition receptors (PRRs), which identify viral components and trigger immune responses, host cells can identify invasive RNA viruses such as VSV [70]. Membrane-associated PRRs that sense VSV infection and initiate antiviral responses include cytosolic receptors, such as RIG-I, and Toll-like receptors (TLR4, TLR3, TLR7, and TLR8). Because cells lacking RIG-I produce fewer type I interferons during VSV infection, RIG-I is especially crucial. Interferon-stimulated genes (ISGs) are produced as a result of these signaling pathways, and many of these genes have antiviral characteristics [70,71]. Membrane fusion, which is a crucial stage in viral entry, is prevented by cholesterol-25-hydroxylase, an ISG. Other ISGs, notably IFITM3 and PKR, block other phases of the VSV lifecycle. The intricate balance between immunological control and immune defense during VSV infection is highlighted by the ability of ISGs to negatively regulate antiviral responses to prevent excessive immune activation [55,72]. The interaction between pathways leading to apoptosis and cell survival is critical for VSV infection. Intrinsic and extrinsic apoptotic pathways are triggered by VSV, which results in caspase- and mitochondria-mediated cell death [73,74]. Apoptosis is largely induced by the matrix (M) protein and leader RNA of the virus, although pro-apoptotic and anti-apoptotic proteins are regulated by biological factors such as hnRNP K, which are crucial for ensuring cell survival [75]. The intricate relationships between viral proteins and host cellular components are highlighted by the capacity of hnRNP K to inhibit apoptosis and promote VSV replication. Interestingly, many cancer cells have elevated levels of hnRNP K, which could contribute to the understanding of why tumor cells facilitate VSV replication [76,77,78].

Furthermore, anti-apoptotic substances, such as Bcl-2, and other cellular proteins that promote apoptosis, such as hnRNPA1, have an impact on VSV-mediated apoptosis. These dynamics demonstrate how VSV modifies host cell survival processes to its benefit, either by inducing apoptosis to promote viral dissemination or by encouraging cell survival to guarantee reproduction. All these pathways together show the complex interplay between host variables that VSV uses to both replicate and survive in the host [79,80]. Autophagy is an important cellular activity identified during VSV infection. Although autophagy is primarily involved in the degradation of undesirable intracellular components, it also plays a dual role in viral infection. Autophagy has an antiviral function in VSV, particularly in Drosophila models. TLR-7-mediated recognition of the VSV glycoprotein activates the autophagy pathway, emphasizing the function of the glycoprotein in the host’s immunological response. Nevertheless, depending on the type of virus, autophagy may be a “necessary evil” for the host, because some viruses, such as Dengue and Hepatitis C, use autophagy to their advantage [55,81,82].

## 7. Transmission and Clinical Manifestations

VSV transmission occurs via various routes, including insect vectors and direct contact with infected animals. It is critical to distinguish natural transmission routes, such as direct animal contact or insect vectors, from clinical inoculation methods employed in research and vaccine studies, such as intranasal, intradermal, and intravenous routes. For example, clinical inoculations, such as intranasal or intravenous methods, are primarily used in experimental settings to study infection mechanisms or for vaccine administration and are not natural transmission routes [83]. These clinical inoculation routes may result in specific clinical manifestations such as fever, flu-like symptoms, or transient viremia in laboratory animals or human participants, which differ from symptoms in livestock naturally infected through insect vectors or animal contact [83]. In natural settings, VSV transmission occurs through direct contact or insect bites, with clinical manifestations typically present in animals as vesicular lesions on mucocutaneous junctions, such as the mouth, nostrils, coronary bands, and udders. These vesicles can progress into ulcers, causing lameness, discomfort, and sometimes, weight loss or reduced milk production in dairy cattle. In horses, lesions tend to affect the tongue, lips, and gums, causing salivation and swallowing difficulties. These symptoms are typically self-limiting, with healing occurring within two–three weeks, although secondary bacterial infections may develop if the lesions are not properly managed [83]. Subclinical infections are also common, and up to 90% of animals in endemic herds may be seropositive without showing clinical signs, indicating the potential role of subclinically infected animals as reservoirs for insect vector transmission. Insect vectors, including blackflies, sandflies, and mosquitoes, have been shown to transmit VSV, with blackflies showing competence in transmitting the virus. This suggests the possibility of virus maintenance in nature through mechanisms such as the co-feeding of infected and uninfected flies on non-viremic hosts [84]. Pathogenesis studies on laboratory animals, such as rodents, often show viremia associated with encephalitis or meningitis, which is not typically observed in infected livestock. In livestock, VSV lesions generally remain localized at the inoculation site, with systemic spread more commonly documented in rodent models. For instance, studies in horses have demonstrated undetectable VSV antigen presence in late disease stages, underscoring the differences in immune responses across species [83,85].

The clinical manifestations of VSV vary significantly depending on the host species, inoculation route, and viral strain. The production of vesicles or blister-like lesions on mucocutaneous junctions, especially around the mouth, nostrils, coronary bands, and udder, is a typical clinical symptom in animals including cattle, horses, and pigs [86]. These lesions frequently develop into ulcers, which can cause discomfort, lameness, and unwillingness to eat. In dairy cattle, this can result in weight loss and reduced milk output. VSV lesions usually affect the tongue, lips, and gums in horses; they can also cause salivation and difficulty swallowing. Lesions typically heal within two three weeks, indicating that the condition is largely self-limiting. However, if ulcers are not well treated, secondary infections may develop [83,87,88]. Subclinical infections are common, particularly in endemic areas. As previously noted, up to 90% of animals harbor antibodies against VSV without exhibiting any obvious clinical symptoms [89]. In these situations, the virus may be maintained and spread throughout the population by infected animals acting as reservoirs. The inability to identify infected animals solely by their clinical signs makes the subclinical form of the disease more difficult to detect and contain VSV outbreaks [88]. Occasionally, VSV may produce flu-like symptoms in humans such as fever, headaches, and muscle aches. Rarely, more serious symptoms such as encephalitis may appear, especially in people with weakened immune systems. However, human VSV infections are usually moderate and self-limiting, with no long-term consequences [90]. VSV outbreaks can have a substantial financial impact on cattle, as quarantines and mobility restrictions are frequently implemented to stop the virus from spreading. Trade restrictions may result from these actions, especially in areas where VSV is not common. The ephemeral nature of the illness and the problem of detecting subclinical infections make it difficult to manage and control the disease in impacted areas [91,92,93].

## 8. Technological Innovations in Oncolytic VSV Development

### 8.1. Oncolytic Virotherapy: Principles and Mechanisms

Oncolytic virotherapy represents a groundbreaking approach to cancer treatment, leveraging the natural propensity of viruses to infect and lyse cells [94]. Owing to its unique properties, the VSV has emerged as a promising candidate in this field. The fundamental principle of VSV-based oncolytic therapy relies on the ability of the virus to selectively replicate in and destroy cancer cells, while sparing normal tissues [95,96]. This selectivity stems from defective interferon responses commonly observed in cancer cells, which render them more susceptible to viral infections. VSV exhibits several mechanisms of tumor cell death. Primarily, it induces direct oncolysis through rapid viral replication, leading to cell lysis and release of tumor-associated antigens [97]. This process not only destroys cancer cells but also stimulates a robust antitumor immune response. Additionally, VSV infection triggers apoptotic pathways in cancer cells, further enhancing its therapeutic efficacy [98]. The ability of the virus to spread rapidly through tumor tissues, coupled with its capacity to induce immunogenic cell death, makes it a potent oncolytic agent. One of the key advantages of using VSV as an oncolytic virus is its rapid replication cycle, which allows efficient tumor cell lysis before the host immune system can mount a significant antiviral response [9]. Furthermore, VSV possesses inherent immune evasion mechanisms such as the ability to suppress host cell protein synthesis, which contributes to its oncolytic potential [99]. The low prevalence of pre-existing immunity to VSV in human populations is another significant advantage as it reduces the risk of rapid viral clearance upon administration [100].

### 8.2. Engineering VSV for Oncolytic Therapy

Various genetic modifications have been introduced to enhance the tumor selectivity and safety profiles of VSV. VSV gene expression is controlled by viral promoter sequences that are specifically recognized by viral RNA-dependent RNA polymerase [101]. Therefore, tumor selectivity is achieved through modifications of viral genes and their natural regulatory elements, rather than through tissue-specific promoters. For instance, one study developed a recombinant VSV expressing the interferon-β gene (VSV-IFNβ), which selectively replicates in and kills tumor cells with defective interferon signaling pathways [102,103]. This modification not only enhances tumor specificity but also improves the safety profile of the virus by limiting its replication in normal cells. Another strategy to improve VSV oncoselectivity involves the deletion or modification of viral virulence genes. A VSV variant with a mutated matrix protein (M51R) has been reported to exhibit enhanced tumor selectivity and reduced neurotoxicity in animal models [104]. This modification attenuates the ability of the virus to shut down host cell gene expression, making it less pathogenic to normal cells while maintaining its oncolytic potential in cancer cells. Researchers have engineered VSVs to express various therapeutic transgenes to further enhance the efficacy of VSV-based oncolytic therapy. For example, a study developed a VSV expressing the herpes simplex virus thymidine kinase (HSV-TK) gene, which sensitizes tumor cells to the antiviral drug ganciclovir, providing an additional layer for tumor-specific killing [105]. Additionally, VSV has been engineered to express immunostimulatory cytokines, such as interleukin-15 (IL-15), to enhance antitumor immune responses [106]. Preclinical studies have demonstrated the efficacy and safety of engineered VSV variants using various cancer models. A previous study showed that VSV-IFNβ effectively suppressed tumor growth in mouse models of hepatocellular carcinoma and colorectal cancer [104]. Another study reported that VSV expressing IL-15 significantly enhanced survival in a murine model of metastatic colon cancer [107].

### 8.3. Clinical Applications and Trials

Several clinical trials have evaluated the safety and efficacy of VSV-based oncolytic therapies in cancer patients (Table 3). A significant challenge in the clinical translation of VSV-based therapies is the inherent neurotropism and potential neurotoxicity of the virus [108]. To address this safety concern, researchers have developed attenuated VSV variants, including VSV-IFNβ-NIS, which incorporate interferon-β to protect normal neural tissue while maintaining oncolytic activity against cancer cells with defective interferon responses [102]. A phase I trial of VSV-IFNβ-NIS, a recombinant VSV expressing both interferon-β and sodium iodide symporter (NIS), demonstrated safety and preliminary efficacy in patients with advanced solid tumors [102]. This trial showed that the intravenous administration of VSV-IFNβ-NIS was well tolerated and resulted in viral replication within tumors, as evidenced by NIS-mediated radioiodine uptake. The incorporation of interferon-β into this variant serves a dual purpose: protecting normal neural tissue from viral infection while maintaining therapeutic efficacy against cancer cells. In another phase I study, VSV-hIFNβ was evaluated in patients with hepatocellular carcinoma [109]. This trial demonstrated the safety of intratumoral administration of VSV-hIFNβ and provided evidence of its antitumor activity in some patients. Notably, the study reported the induction of systemic immune responses against tumor antigens, suggesting the potential of VSV to stimulate anti-tumor immunity. Despite these promising results, several challenges remain in the clinical application of VSV-based oncolytic therapies. A critical consideration is achieving an optimal balance between maintaining oncolytic efficacy and minimizing neurotoxicity, particularly when treating tumors near neural tissues. One major hurdle is the potential for premature viral clearance by the host immune system, which can limit the efficacy of treatment [110]. Researchers are exploring various strategies to address this issue, including methods to enhance circulation time and tumor accumulation [105]. To address this issue, researchers are exploring strategies such as the polymer coating of viral particles to enhance the circulation time and tumor accumulation [105]. Another challenge is the development of resistance mechanisms in tumor cells. Some cancer cells may upregulate antiviral pathways or develop mutations that confer resistance to VSV infection [111,112]. Combination strategies, such as combining VSV with immune checkpoint inhibitors or other immunotherapies, are being investigated to overcome these resistance mechanisms and enhance the overall therapeutic efficacy [113,114]. The future of VSV-based oncolytic therapies appears promising. Ongoing research is focused on developing personalized approaches that combine VSV with patient-specific tumor antigens or neoepitopes to enhance antitumor immune responses [104]. Additionally, the potential of VSV to serve as a vector for cancer vaccination strategies is being explored, opening new avenues for cancer immunotherapy [9].

## 9. Technological Innovations in VSV-Based Vaccine Development

### 9.1. VSV as a Vaccine Vector

VSV has emerged as a promising vaccine vector owing to its unique characteristics and adaptability [4]. The VSV platform offers several advantages, including rapid replication, robust immune activation, and versatility in accommodating various pathogen-specific antigens [9]. These properties make VSV an attractive candidate for vaccine development for a wide range of infectious diseases. One of the key strengths of VSV as a vaccine vector is its ability to induce both humoral and cellular immune responses [118]. When used as a vaccine platform, VSV can stimulate the production of neutralizing antibodies and activate T cell responses, providing comprehensive protection against target pathogens [119]. This dual-action immune stimulation is particularly valuable for the development of vaccines for complex diseases that require multifaceted immune responses. Genetic engineering techniques have been used extensively to optimize VSV for vaccine development. Researchers have successfully inserted pathogen-specific antigens into the VSV genome, allowing for the expression of foreign proteins on the viral surface [120]. For instance, the incorporation of HIV-1 Envelope (Env) proteins into VSV particles has been achieved with modifications to enhance surface expression and immunogenicity [121]. Additionally, safety modifications have been implemented, such as attenuating the virus to reduce its pathogenicity, while maintaining its immunogenic properties [122]. A notable case study on VSV-based vaccine development is the Ebola virus vaccine rVSV-ZEBOV [10]. The vaccine was deployed during the tenth ebolavirus outbreak in the Democratic Republic of the DRC under the expanded access framework [123]. Preliminary unadjusted analyses estimated the vaccine effectiveness to be 98% (95% CI 96–99) [124]. The success of rVSV-ZEBOV has paved the way for adapting the VSV platform to other pathogens including SARS-CoV-2. In the context of COVID-19, researchers have rapidly developed VSV-based vaccine candidates expressing the SARS-CoV-2 spike protein [125]. One such candidate, VSV-SARS2-EBOV, combines the SARS-CoV-2 spike protein with the Ebola virus glycoprotein [126]. Preclinical studies in rhesus macaques have shown that a single intramuscular dose of this vaccine provided protection against COVID-19 pneumonia within 10 days of administration [127].

### 9.2. Vaccine Development Strategies

Various strategies have been developed to enhance the immunogenicity of VSV-based vaccines. One approach involves the use of chimeric antigens to optimize antigen presentation and increase immune response. For example, researchers have developed HIV-1 Env chimeras incorporating the transmembrane domain and cytoplasmic tail of SIVMac239, resulting in a higher surface expression of VSV particles [128]. This modification has led to significantly improved antibody responses in animal studies. Prime-boost vaccination protocols have been explored for enhancing the efficacy of VSV-based vaccines. In one study, a VSV-HIV prime followed by a DNA boost strategy was investigated in rhesus macaques [129]. Although this approach induced non-neutralizing antibody responses and systemic memory T-cell activation, it did not provide observable protection against SHIV infection in an animal model [129].

Efforts to enhance vaccine stability and delivery have focused on developing temperature-stable formulations and advanced delivery methods. For VSV-based vaccines, research has commonly explored lyophilization techniques and nanoparticle-based formulations to improve both stability and delivery efficiency [130,131]. Compared with other vaccine platforms, such as adenovirus-based and mRNA vaccines, VSV-based vaccines have shown promising results in terms of rapid immune response induction and single-dose efficacy [132]. This rapid immunogenicity makes VSV-based platforms highly suitable for outbreak settings where time-sensitive responses are critical. Additionally, the single-dose efficacy observed with VSV-based vaccines reduces the logistical challenges associated with multi-dose regimens, which is a limitation of some adenovirus-based and mRNA vaccines [133].

Although effective, adenovirus-based vaccines often require more than one dose to achieve long-lasting immunity, and their efficacy can be influenced by pre-existing immunity to the viral vector used, potentially diminishing their effectiveness [134,135]. mRNA vaccines, although groundbreaking in their ability to deliver genetic instructions for antigen production, typically require two doses for optimal protection and rely heavily on cold chain storage, which presents a challenge in resource-limited settings [136,137]. The safety profile of VSV-based vaccines has generally been favorable, as evidenced by the approval of rVSV-ZEBOV for human use [116]. However, it is important to acknowledge that the route of vaccine administration significantly influences both the safety and efficacy. In a study evaluating the VSV-SARS2-EBOV vaccine, intramuscular administration demonstrated protective effects without inducing symptoms of COVID-19 pneumonia. In contrast, intranasal administration resulted in limited immunogenicity and was associated with an increased incidence of COVID-19 pneumonia compared with that in the control group [132]. VSV-based vaccines are promising for addressing a wide range of pathogens, including HIV, influenza, and emerging viruses [128,129,138]. The versatility of this platform and its rapid production capabilities make it a valuable tool for pandemic preparedness and response.

## 10. Current Challenges in VSV Applications

### 10.1. Technical and Scientific Challenges

Engineering and generating viruses for therapeutic and scientific applications present various hurdles that can hamper advancements in virology, gene therapy, and vaccine development. Genetic stability is an important consideration in viral engineering. Viruses undergo mutations as they replicate, which may result in unanticipated behavioral or pathogenic alterations. This may complicate the production of modified viruses intended for therapeutic use, as advantageous mutations may unintentionally lead to more virulent strains, posing dangers to safety and efficacy [139]. Robust quality control procedures are crucial for viral engineering. Variability in assembly quality can result in chimeric sequences or partial viral genomes, compromising the reliability of the created viruses [140]. Establishing community-wide standards for viral genome assembly is critical to ensure the accuracy and reliability of modified viruses used in research and therapy [140]. Purifying viral vectors after manufacturing presents a substantial difficulty. Contaminants can compromise the safety and efficacy of the end products. Developing efficient purification procedures that can handle the complexity of viral preparations while maintaining the integrity of viral particles is vital for successful applications in gene therapy and vaccine manufacturing [141].

Understanding and predicting viral evolution is a difficult task, especially for RNA viruses that have tremendous mutation rates. The problem is the development of experimental systems that accurately reflect viral development in natural environments [142]. Laboratory experiments frequently fail to mimic the selective forces present in vivo, resulting in inconsistencies between the created viruses and their wild-type counterparts [142]. Another challenge is to effectively deliver modified viruses. The delivery route has a substantial impact on the immune response and therapeutic efficacy of the virus. Developing tailored delivery systems that can cross the immune system and reach specific regions is critical for maximizing the potential of modified viruses [143]. Overcoming safety concerns in virus engineering and production is crucial, particularly considering the potential risks of altering dangerous viruses. The dual-purpose potential of viral engineering requires ethical consideration. Advances in viral vector technology may be misused to create diseases with pandemic potential [144]. This requires strong regulatory control and ethical rules to prevent misuse while stimulating innovation in virus engineering for positive purposes [143,144].

### 10.2. Regulatory and Ethical Considerations

The regulatory and ethical landscape for oncolytic and vaccination therapy is complicated and requires careful navigation to ensure patient safety and adherence to legal requirements. These guidelines ensure the safety and efficacy of clinical research, while also safeguarding participants’ rights. Oncolytic therapies must adhere to stringent regulations, such as EU Regulation 536/2014, which mandates scientific authorization and ethical approval from Research Ethics Committees (RECs) [145]. Vaccine development includes thorough preclinical and clinical tests to assess safety and efficacy, along with continued post-licensure surveillance to manage adverse events [146]. Ethical frameworks emphasize informed consent, transparency, and respect for autonomy, especially in vulnerable populations, such as children [146,147]. The quality of clinical trial protocols is crucial, and ethical reviews often highlight the need for clear, respectful communication regarding patient rights and safety [145]. While regulatory compliance is critical, ethical considerations must also prioritize participant welfare and informed consent to ensure that research benefits public health while protecting individual rights.

### 10.3. Other Challenges Related to VSV

In addition to vaccine development and oncolytic therapy, VSV infection poses several challenges. These drawbacks limit their usefulness as research tools and therapeutic agents. One of the most significant obstacles to using VSV as a therapeutic vector is the immunological response of the host. The immune system can recognize and respond to viral vectors, which may restrict the efficacy of subsequent treatments [9]. Overcoming these immunological obstacles is critical for improving the therapeutic potential of VSV-based vectors, particularly in gene therapy and oncolytic virotherapy [9]. The emergence of viral escape mutants is another source of concern. These mutations can emerge during therapy, potentially resulting in breakthrough infections and diminished efficacy of vaccination or oncolytic medicines. To reduce this risk and ensure long-term efficacy against targeted diseases, VSV vectors must be continuously monitored and adjusted [148].

Scaling up the manufacturing of VSV for clinical use presents considerable obstacles. The availability of the virus for research and therapeutic uses may be limited by the complexity of its manufacturing methods, including the need for high containment facilities owing to its pathogenic nature [9]. The advancement of VSV-based products requires efficient production techniques that provide high yield and quality [9]. VSV is predominantly an agricultural virus, and its use as a vaccine vector raises concerns regarding its pathogenicity in non-target animals, such as livestock and humans [4]. For example, high doses of VSV-vectored vaccinations have been linked to vesicular illnesses in pigs, emphasizing the necessity for rigorous consideration of safety profiles in various populations [4]. It is possible that altered VSV will return to a more virulent form, especially if the virus is attenuated for medicinal purposes. This reversion may present safety hazards, demanding comprehensive evaluations of the stability and behavior of recombinant VSV in clinical settings [4,94]. Although VSV has great potential as a therapeutic vector and research tool, overcoming these difficulties is critical to its effective use in medicine. Continued research and innovation in viral engineering, immunology, manufacturing methods, and regulatory frameworks are required to realize its full potential.

## 11. Future Research Directions

Future research directions for VSV include evolving technology, novel uses, and the possibility of combination therapy and multitarget strategies. The combination of gene-editing technologies, such as CRISPR-Cas9 and VSV-based vectors, shows potential for targeted gene therapy [9,149]. This combination may increase the precision of genetic changes and improve the therapeutic outcomes for genetic disorders and malignancies. Emerging technologies such as artificial intelligence (AI) and machine learning have been used to transform the examination of VSV data [150]. Using big data analytics, researchers can better understand viral behavior, optimize vector design, and anticipate outcomes in clinical settings, ultimately improving the development of VSV-based medicines [151]. VSV can be designed to produce nanostructures for various applications, including medication delivery systems and biosensors [152]. Modifying VSV to display certain peptides or proteins allows the development of targeted medicines and diagnostics.

VSV’s potential as an oncolytic virus can be increased by combining it with other treatments, such as immune checkpoint inhibitors or conventional chemotherapy [9]. This multi-target approach may result in synergistic effects that improve tumor response rates and patient outcomes. Combining VSV with different viral vectors or adjuvants may increase vaccination effectiveness. For example, employing VSV as a platform for delivering antigens from many pathogens could provide comprehensive protection against infectious diseases, including emerging viral threats [9]. The combination of VSV-based medicines and personalized medicine approaches helps adapt treatments to specific patient characteristics. The use of biomarkers to guide the selection of VSV vectors and combination medicines may improve treatment success and minimize side effects [9,150]. The future of VSV research is expected to benefit from technological development and novel therapeutic options. Researchers can unlock new possibilities for VSV as a therapeutic tool by investigating emerging technologies and adopting combination therapies.

## 12. Conclusions

This study on VSV provides crucial insights into its pathophysiology and immune evasion mechanisms, shedding light on how the virus can elude host defenses while specifically targeting cancer cells. Understanding VSV’s fundamental biology, particularly the use of LDL-R for cell entry and reliance on its viral promoter sequences, has been crucial in developing more effective therapeutic strategies. Technological developments in VSV engineering have resulted in potential advances in both oncolytic therapy and vaccine development, thereby demonstrating its versatility as a therapeutic platform. The successful attenuation of VSV neurotropism, while maintaining its oncolytic potential, represents a significant achievement in its development as a therapeutic agent. VSV’s unique features not only increase its potential as an oncolytic drug but also make it an efficient vaccination vector for emerging infectious illnesses. The capacity to elicit strong immune responses while maintaining a low-risk profile is a significant advantage in public health applications.

Despite its promise, problems such as genetic stability, effective delivery systems, and regulatory impediments must be overcome to fully realize VSV’s therapeutic potential. Particular attention must be paid to managing the inherent neurotropism of the virus while maintaining its therapeutic efficacy. Continued research is required to optimize VSV for clinical use and ensure its efficacy and safety across various populations. VSV stands out as a transformative tool in medicine, and ongoing research is expected to open new pathways for its use in cancer treatment and infectious disease control. Emphasizing collaborative research, ethical considerations, and new techniques is critical in leveraging VSV’s strengths to improve global health outcomes.

## Figures and Tables

**Table 1:** VSV structure, serotype, virion features, entry and replication mechanisms, and interactions with host cells.

Feature	Details
Virus Family	Rhabdoviridae
Genus	Vesiculovirus
Structure	Enveloped, nonsegmented, negative-stranded RNA virus
	Bullet-shaped virion structure
	Size: 65 to 185 nm
Main Serotypes	New Jersey (VSNJV)
	Indiana (VSV)
Indiana Serotypes	Indiana type 1 (classical strain)
	Cocal virus (COCV)—subtype 2
	Alagoas virus (VSAV)—subtype 3
Other Vesiculoviruses	Piry virus
	Chandipura virus
	Isfahan virus
Virion Structure	Two main parts: internal ribonucleoprotein core and exterior envelope
	G protein (glycoprotein) on envelope forms ~400 trimeric spikes
	N protein (nucleoprotein) encapsulates RNA in “beads-on-a-string” structure
	L protein (large polymerase protein)
	Matrix protein (M) between nucleocapsid core and envelope supports nucleocapsid condensation and budding
Viral Proteins	Nucleoprotein (N)
	Phosphoprotein (P)
	Matrix protein (M)

**Table 2:** VSV strategies for host manipulation, translation control, transcriptional inhibition, and immune evasion.

Process	Mechanism/Host Interaction	References
Host Translation Machinery Manipulation	VSV uses a cap-dependent translation strategy, selectively prioritizing its mRNA by altering the eIF4F complex and dephosphorylating 4E-BP1 and eIF4E. It employs structural elements in its mRNA that interact with ribosomal proteins like rpL40, aiding viral mRNA translation.	[38,49,50,51]
Host Transcription Inhibition	The VSV M protein interacts with Rae1 and Nup98 to inhibit cellular transcript export. It deactivates TFIID and downregulates RNA polymerase II, blocking cellular transcription.	
Replication Machinery Manipulation	Host factors like tubulin aid in VSV genome replication, and HSP90AB1 stabilizes the viral RNA polymerase. La proteins, interacting with viral RNAs, enhance replication efficiency.	[56,57]
Viral Assembly and Budding	VSV G protein is glycosylated in the ER and transported via COPI/COPII to the plasma membrane, with SEC22b aiding transport. M protein forms microdomains in the plasma membrane, facilitating nucleocapsid assembly and interacting with host proteins like YAP and dynamins.	
Viral Budding and Release	It involves ESCRT machinery and proteins like TSG101, VPS4A/B, and the proteasomal pathway. M protein interacts with lipids and proteins in the plasma membrane to ensure effective budding.	[25,55,58]
Immune Evasion	VSV evades immune responses by modulating apoptosis, inhibiting interferon-stimulated genes (e.g., cholesterol-25-hydroxylase, IFITM3, PKR), and using apoptotic proteins like Bcl-2 and hnRNPA1 to favor viral replication.	

**Table 3:** Versatile platform for cancer therapy and vaccine development.

Feature	Oncolytic Virotherapy	Vaccine Development	References
Mechanism	Destroys cancer cells directly through lysis and indirectly by stimulating an immune response. VSV selectively replicates in cancer cells due to their defective interferon responses.	Delivers pathogen-specific antigens to trigger an immune response. VSV’s ability to induce both humoral and cellular immune responses makes it effective against various pathogens.	[115]
Engineering	Engineered for enhanced tumor selectivity and safety. This includes using tumor-specific promoters, modifying viral virulence genes, and expressing therapeutic transgenes.	Engineered for optimal antigen presentation, safety, and stability. Scientists have inserted pathogen-specific antigens into the VSV genome and attenuated the virus for safety. Strategies also include chimeric antigens, prime-boost vaccination, temperature-stable formulations, and nanoparticle-based formulations.	[105]
Clinical Applications	Clinical trials show promising results but face challenges. Trials demonstrate safety and efficacy in various cancers, including the induction of antitumor immunity. Challenges include premature viral clearance and tumor resistance.	Successfully used in Ebola vaccine and shows promise for other diseases. rVSV-ZEBOV, an Ebola vaccine, is approved for human use. VSV-based COVID-19 vaccines are under development. Other potential targets include HIV, influenza, and emerging viruses.	[116]
Advantages	Rapid replication, inherent immune evasion mechanisms, and low pre-existing immunity in humans.	Rapid replication, robust immune activation, versatility in accommodating antigens, and single-dose efficacy. Compared to adenovirus-based and mRNA vaccines, VSV offers advantages like rapid immunogenicity and single-dose efficacy.	[117]
Challenges	Premature viral clearance by the immune system and development of resistance in tumor cells. Combination strategies and personalized approaches are being explored to address these.	Route of administration can significantly impact safety and efficacy. While generally safe, VSV-based vaccines’ effectiveness can vary depending on the delivery route.	[117]

## Data Availability

Not applicable because no new data or databases were used in the preparation of this work.
